# Serial cross-sectional data on the public's perception on the coronavirus during the first months of the pandemic in Germany

**DOI:** 10.1016/j.dib.2021.107430

**Published:** 2021-09-25

**Authors:** Fabian Kirsch, Ann-Kathrin Lindemann, Mark Lohmann, Gaby-Fleur Böl

**Affiliations:** German Federal Institute for Risk Assessment (BfR)^2^, Berlin, Germany

**Keywords:** Risk perception, Protective behaviour, Information behaviour, Coronavirus, COVID-19, Survey data, Trend study

## Abstract

The coronavirus pandemic poses major challenges for governments and public health authorities. In order to implement appropriate measures, it is important to understand how the population is coping with the pandemic. This dataset contains serial cross-sectional survey data from the first months of the coronavirus pandemic in Germany. Data were collected between 24 March and 26 May 2020 in ten weekly surveys (*n*s ranging between 500 and 515, in total *N* = 5,076) as part of omnibus telephone interviews. Samples were drawn at random from landline and mobile telephone numbers. The main topics of the questionnaire were (a) the expected impact of the coronavirus on one's personal life, (b) perception of infection risk, (c) protective measures and (d) information behaviour. Data were weighted to ensure sociodemographic representativeness. To account for the rapidly changing situation of the coronavirus pandemic in Germany, the questionnaire underwent several adjustments during the data collection period.

## Specifications Table

**Table:** 

Subject	Social Science
Specific subject area	Expected impact on one's personal life, perception of infection risk, protective measures and information behaviour during the coronavirus pandemic in Germany
Type of data	Tables Raw data (Dataset 1) Coded data (Dataset 2) Questionnaire (Appendix A) Code frames (Appendix B)
How data were acquired	Data were obtained via a ten-wave telephone survey during the first months of the coronavirus pandemic in Germany (March – May 2020). Samples were drawn at random from landline and mobile telephone numbers. An overview of the questionnaire is included in Appendix A.
Data format	Raw Coded
Parameters for data collection	German-speaking population aged 14 years and over in private households in the Federal Republic of Germany who could be reached via mobile or landline telephone numbers
Description of data collection	Data were collected by a marketing research institute as part of omnibus telephone surveys. Between 24 March and 26 May 2020, about 500 randomly selected people were interviewed every week. Topics of interest were (a) the expected impact of the coronavirus on one's personal life, (b) perception of infection risk, (c) protective measures and (d) information behaviour. The complete dataset contains survey data of 5,076 people. Data were weighted to ensure sociodemographic representativeness. Due to the rapidly changing situation regarding the coronavirus pandemic, the questionnaire underwent several adaptations over the course of data collection.
Data source location	Institution: German Federal Institute of Risk Assessment (BfR) City/Town/Region: Berlin Country: Germany
Data accessibility	With the article

## Value of the Data

• The recurring assessment of the public perception at the onset of a pandemic can provide crucial insights for the management of future pandemics or other crises by providing data on how quickly the population adopts protection measures or their acceptance of different containment measures.
• In-depth analysis of the data can aid public health authorities in drafting risk communication measures tailored to different target audiences like the elderly, who are particularly at risk from this virus.
• The data provide a base for secondary analyses in terms of comparisons with infection rates, media coverage, perceptions across other countries and over the course of the first months of the pandemic.
• The data provide insights in a broad variety of the public's experiences and perceptions by considering diverse thematic areas of interest (impact on one's personal life, perception of infection risk, protective measures, information behaviour).

## 1 Data Description

We conducted a series of surveys with the aim to capture the population's perception of what is happening around the coronavirus over time. More specifically, we collected data on (a) the expected impact of the coronavirus on one's personal life, (b) perception of infection risk, (c) protective measures and (d) information behaviour (see Appendix A for an overview of the questionnaire). For all of these thematic areas, our questionnaire considers specific important aspects within the pandemic without any claim to completeness.

The presented data were collected during the first months of the coronavirus pandemic in Germany. Data were collected via telephone interviews in 10 survey waves between 24 March and 26 May 2020 (total *N* = 5,076). Data were weighted based on socio-demographic characteristics for each individual wave to ensure representativeness. All presented results are based on weighted data. Table 1 provides an overview of the survey waves, including the date of data collection, corresponding sample sizes and key sociodemographic variables.

**Table 1:** Overview of survey waves and key sociodemographic variables.

	Total	W1	W2	W3	W4	W5	W6	W7	W8	W9	W10
**Overview**											

date (year 2020)	–	24.03.	31.03.	07.04.	14.04.	21.04.	28.04.	05.05.	12.05.	19.05.	26.05.
*n*	*5,076*	*510*	*500*	*510*	*515*	*505*	*503*	*504*	*510*	*509*	*510*

**Gender**											

male (%)	49.1	49.6	48.6	49.0	48.6	48.8	48.9	48.7	50.1	48.6	50.1
female (%)	50.9	50.4	51.4	51.0	51.4	51.2	51.1	51.3	49.9	51.4	49.9

**Age**											

*M* (years)	49.7	48.8	50.2	50.0	49.6	49.7	49.6	49.6	50.3	48.9	50.4
*SD* (years)	19.5	19.8	20.1	19.8	19.4	19.4	19.4	19.8	18.9	19.3	19.3

**Education**											

pupil (%)	3.8	4.2	3.4	4.0	4.3	3.7	3.6	3.8	3.4	4.2	3.4
secondary general school (Volks-/ Hauptschule) (%)	33.3	34.3	33.3	33.2	32.4	32.0	34.2	33.8	31.6	33.9	34.5
secondary school without Abitur (%)	30.0	29.5	30.2	29.6	29.5	30.0	29.8	29.8	31.0	30.2	29.9
Abitur, university/polytechnic entrance qualification (%)	14.2	16.6	13.7	13.7	12.1	18.4	12.3	16.0	14.8	12.7	12.0
academic degree (university, academy, polytechnic) (%)	18.7	15.4	19.4	19.6	21.7	15.9	20.0	16.7	19.2	19.0	20.2

**Size of city**											

up to less than 20,000 inhabitants (%)	13.2	13.2	13.8	12.7	12.8	12.6	12.5	13.3	13.6	13.5	13.7
20,000 to less than 100,000 inhabitants (%)	20.9	21.1	21.4	20.5	20.7	21.0	20.4	21.2	20.6	20.9	21.1
100,000 to less than 500,000 inhabitants (%)	29.5	29.9	28.3	30.5	30.6	30.6	30.2	29.3	28.0	29.2	28.4
500,000 inhabitants and more (%)	36.4	35.9	36.4	36.3	35.9	35.9	36.9	36.2	37.9	36.4	36.7

W = wave.

Dataset 1 contains the raw, unprocessed data including the paraphrased answers to the open-ended questions. All variable and value labels as well as the paraphrased answers are in German language. In Dataset 2, the variable and value labels have all been translated to English and the paraphrased answers were coded using the code frames accessible under Appendix B.

Table 2 displays data on the expected impact on one's personal life. The table contains the descriptive statistics on two survey questions. One question aimed at comparing the perceived health impact of a coronavirus infection with other diseases (cancer and flu). Starting in wave 4, a second question asked the respondents to compare the perceived impact of the coronavirus on their health versus their economic situation.

**Table 2:** Data on the expected impact on one's personal life.

	Total	W1	W2	W3	W4	W5	W6	W7	W8	W9	W10
**Assuming you have one of the following diseases: How large or small do you consider the health effects of this to be for you personally?** Rating question using a response scale of 1 ‘very small’ – 5 ‘very large’											

**Coronavirus disease**											
*n*	*4,888*	*503*	*472*	*485*	*512*	*480*	*485*	*487*	*493*	*483*	*487*
*M*	2.84	2.99	2.94	3.19	2.69	2.88	2.72	2.74	2.71	2.99	2.60
*SD*	1.38	1.50	1.40	1.38	1.33	1.37	1.39	1.35	1.32	1.36	1.33
**Cancer**											
*n*	*4,824*	*497*	*471*	*490*	*498*	*464*	*484*	*482*	*472*	*492*	*472*
*M*	3.61	3.60	3.61	3.63	3.39	3.68	3.44	3.68	3.64	3.73	3.75
*SD*	1.43	1.54	1.46	1.37	1.48	1.35	1.48	1.45	1.34	1.38	1.37
**Flu**											
*n*	*4,993*	*508*	*482*	*491*	*513*	*492*	*501*	*497*	*505*	*506*	*497*
*M*	2.17	2.32	2.37	2.27	2.11	2.02	2.03	2.09	2.22	2.15	2.13
*SD*	1.15	1.33	1.20	1.15	1.13	1.08	1.02	1.09	1.13	1.20	1.13

**What do you think affects you more: the impact of the novel coronavirus on health or on the economic situation?**Single selection question											

*n*	*3,529*	*–*	*–*	*–*	*515*	*499*	*502*	*497*	*509*	*506*	*499*
impact on health (%)	21.9	–	–	–	24.0	21.3	30.6	21.7	21.5	15.3	19.2
impact on economic situation (%)	35.2	–	–	–	35.9	36.0	28.5	34.1	33.9	38.7	39.3
both equally (%)	29.0	–	–	–	27.2	31.8	29.2	26.5	31.0	32.0	25.1
neither (%)	13.9	–	–	–	13.0	10.9	11.7	17.7	13.6	13.9	16.5

W = wave; only valid responses were included in the analyses.

Table 3 shows an overview on the collected data on people's perception of their infection risk. The first question assessed the perceived controllability of an infection. The second question addressed the expected probability for an infection via various transmission pathways.

**Table 3:** Data on perception of infection risk.

	Total	W1	W2	W3	W4	W5	W6	W7	W8	W9	W10
**How sure are you that you can protect yourself from an infection with the novel coronavirus?**Rating question using a response scale of 1 ‘not sure at all’ – 5 ‘very sure’											

*n*	*5,007*	*499*	*494*	*509*	*514*	*496*	*497*	*492*	*504*	*501*	*500*
*M*	3.05	2.79	3.02	2.87	3.01	3.14	3.13	3.24	3.24	3.06	3.04
*SD*	1.21	1.29	1.20	1.19	1.28	1.16	1.12	1.11	1.21	1.23	1.19

**How high or low do you estimate the probability of being infected with the novel coronavirus via the following paths?**Rating question using a response scale of 1 ‘very low’ – 5 ‘very high’											

**Proximity to other people**											
*n*	*5,031*	*505*	*498*	*503*	*510*	*502*	*500*	*500*	*509*	*509*	*494*
*M*	3.99	4.28	4.20	4.14	4.06	4.07	4.04	3.78	3.90	3.72	3.70
*SD*	1.13	1.00	1.06	1.15	1.12	1.06	1.07	1.14	1.19	1.19	1.15
**Door handles**											
*n*	*5,033*	*502*	*500*	*503*	*511*	*499*	*500*	*503*	*510*	*500*	*506*
*M*	3.34	3.81	3.78	3.36	3.21	3.36	3.16	3.23	3.21	3.29	3.05
*SD*	1.37	1.20	1.27	1.36	1.43	1.38	1.39	1.39	1.35	1.40	1.35
**Toys**											
*n*	*4,868*	*489*	*486*	*492*	*496*	*474*	*481*	*477*	*491*	*498*	*484*
*M*	2.66	2.95	3.03	2.70	2.47	2.70	2.60	2.52	2.64	2.69	2.27
*SD*	1.37	1.37	1.39	1.36	1.33	1.39	1.40	1.29	1.34	1.40	1.31
**Cash**											
*n*	*5,019*	*505*	*497*	*507*	*510*	*499*	*496*	*497*	*504*	*503*	*501*
*M*	2.80	3.31	3.21	2.94	2.82	2.70	2.63	2.58	2.64	2.71	2.48
*SD*	1.38	1.36	1.38	1.35	1.44	1.38	1.39	1.32	1.27	1.36	1.31
**Dishes and cutlery**											
*n*	*4,996*	*499*	*489*	*502*	*510*	*495*	*496*	*496*	*505*	*506*	*498*
*M*	2.27	2.52	2.61	2.20	2.16	2.23	2.12	2.11	2.31	2.41	2.06
*SD*	1.33	1.44	1.37	1.30	1.36	1.32	1.28	1.25	1.30	1.36	1.22
**Food**											
*n*	*5,007*	*500*	*487*	*504*	*511*	*504*	*493*	*500*	*502*	*508*	*499*
*M*	2.05	2.08	2.29	2.13	2.03	2.06	2.00	2.08	1.93	2.04	1.84
*SD*	1.15	1.20	1.22	1.15	1.20	1.13	1.01	1.16	1.05	1.22	1.12
**Pets**											
*n*	*4,822*	*484*	*482*	*472*	*489*	*487*	*477*	*470*	*492*	*490*	*479*
*M*	1.74	1.75	1.83	1.91	1.68	1.79	1.67	1.68	1.71	1.78	1.58
*SD*	1.09	1.13	1.13	1.22	1.09	1.13	0.99	0.98	1.02	1.12	0.98
**Clothing**											
*n*	*4,965*	*499*	*485*	*498*	*510*	*490*	*493*	*493*	*504*	*499*	*493*
*M*	1.90	2.10	2.04	1.88	1.79	1.97	1.84	1.89	1.85	1.88	1.74
*SD*	1.07	1.15	1.10	1.08	1.05	1.09	0.99	1.07	1.01	1.05	0.99

W = wave; only valid responses were included in the analyses.

Tables 4 and 5 contains data on protective measures. Table 4 displays data on the protection measures utilized by the respondents. The respondents were asked whether they have taken measures to protect themselves or their family from the coronavirus, and, if so, which measures they have taken. In the first three waves of data collection, the questionnaire contained an additional question on people's preferences when cleaning their hands (using soap and water versus using disinfectant), which was dropped in wave 4. Table 5 provides data on the respondents’ acceptance on governmental measures. Respondents were presented with items describing the current containments measures in Germany and were asked to indicate if they found those to be appropriate or not. During the data collection period, items of this question had to be adapted several times due to the changes in regulation to guarantee a valid data collection.

**Table 4:** Data on protective measures: Behaviour.

	Total	W1	W2	W3	W4	W5	W6	W7	W8	W9	W10
**Have or had you taken measures to protect yourself or your family from the novel coronavirus?**Single selection question with open-ended response option											

*n*	*5,044*	*504*	*499*	*508*	*515*	*502*	*497*	*504*	*505*	*505*	*506*
no	22.9	32.4	22.4	21.8	25.7	18.9	22.0	21.7	19.0	20.0	24.8
yes, that is …											
hygienic measures (%)	24.4	26.2	30.0	25.5	26.0	30.4	27.8	18.1	19.3	22.5	18.8
protective clothing (%)	27.8	5.3	16.1	22.3	22.5	32.9	36.9	34.3	40.4	33.4	34.5
reduction of contacts (%)	48.5	48.6	48.5	54.8	44.9	50.4	47.4	47.1	54.8	50.8	37.9
keeping physical distance (%)	15.7	12.7	9.4	9.4	13.1	13.0	16.0	17.7	25.8	20.6	19.2
adjusted consumer behaviour (%)	6.7	3.8	6.7	6.2	4.2	10.0	7.9	5.5	8.6	8.7	5.0
compliance with orders and recommendations in general (%)	9.2	4.9	12.3	10.0	8.6	8.7	6.2	11.7	7.8	7.3	14.3
other (%)	6.2	5.9	2.7	7.7	7.7	5.0	5.3	6.2	7.1	7.1	6.9

**If you had to choose, would you rather clean your hands with soap and water or with disinfectant to protect yourself from the novel coronavirus?**Single selection question											

*n*	*1,508*	*503*	*497*	*508*	–	–	–	–	–	–	–
soap and water (%)	82.7	83.8	80.8	83.5	–	–	–	–	–	–	–
disinfectant (%)	17.3	16.2	19.2	16.5	–	–	–	–	–	–	–

W = wave; only valid responses were included in the analyses.

**Table 5:** Data on protective measures: Acceptance.

	Total	W1	W2	W3	W4	W5	W6	W7	W8	W9	W10
**How do you evaluate the following measures to contain the spread of the novel coronavirus?**Single selection question											

**The closure of day-care centres and schools**											
*n*	*3,477*	*504*	*495*	*504*	*509*	*493*	*484*	*488*	–	–	–
appropriate (%)	85.4	94.2	93.1	86.8	89.2	83.4	75.6	75.2	–	–	–
not appropriate (%)	14.6	5.8	6.9	13.2	10.8	16.6	24.4	24.8	–	–	–

**The closure of cultural institutions like cinemas, theatres or museums (W1–W6) / The closure of cultural institutions like cinemas or theatres (W7–W10)**											

*n*	*4,998*	*507*	*500*	*505*	*509*	*496*	*491*	*490*	*509*	*497*	*495*
appropriate (%)	86.7	97.0	96.4	92.7	92.5	89.7	84.9	80.4	76.5	78.9	77.8
not appropriate (%)	13.3	3.0	3.6	7.3	7.5	10.3	15.1	19.6	23.5	21.1	22.2

**The closure of most shops (W1–W4) / The closure of certain shops (W5–W6)**											

*n*	*2,963*	*505*	*488*	*498*	*499*	*489*	*483*	–	–	–	–
appropriate (%)	73.9	86.1	83.6	71.6	64.3	71.2	66.3	–	–	–	–
not appropriate (%)	26.1	13.9	16.4	28.4	35.7	28.8	33.7	–	–	–	–

**The cancellation of events such as fairs, religious services or sporting events (W1–W5) / The cancellation of most events (W6–W10)**											

*n*	*5,006*	*506*	*499*	*501*	*506*	*497*	*497*	*486*	*509*	*499*	*506*
appropriate (%)	91.9	96.9	96.5	96.3	95.4	94.2	87.7	89.1	85.9	86.7	89.7
not appropriate (%)	8.1	3.1	3.5	3.7	4.6	5.8	12.3	10.9	14.1	13.3	10.3

**The implementation of border controls**											

*n*	*4,948*	*506*	*489*	*501*	*507*	*491*	*488*	*483*	*503*	*492*	*488*
appropriate (%)	80.4	91.1	89.9	86.1	83.9	84.7	79.8	76.8	72.2	74.2	65.5
not appropriate (%)	19.6	8.9	10.1	13.9	16.1	15.3	20.2	23.2	27.8	25.8	34.5

**The restriction of travel activities such as air travel**											

*n*	*5,026*	*506*	*496*	*506*	*515*	*500*	*501*	*496*	*506*	*498*	*503*
appropriate (%)	92.0	96.5	96.4	95.0	93.7	96.4	92.9	92.2	85.9	85.1	85.8
not appropriate (%)	8.0	3.5	3.6	5.0	6.3	3.6	7.1	7.8	14.1	14.9	14.2

**The officially ordered quarantine for persons who have had contact with an infected person**											

*n*	*2,018*	*506*	*497*	*505*	*510*	–	–	–	–	–	–
appropriate (%)	95.9	97.0	95.8	96.5	94.4	–	–	–	–	–	–
not appropriate (%)	4.1	3.0	4.2	3.5	5.6	–	–	–	–	–	–

**The contact prohibition, i.e. the almost complete prohibition of groups of more than two people in public (W1–W7) / The contact restriction, i.e. the regulation of how many people one is allowed to meet with (W8–W10)**											

*n*	*4,518*	*503*	*499*	*505*	*514*	*501*	*493*	*496*	*506*	*507*	*498*
appropriate (%)	85.1	91.6	88.2	84.6	84.6	79.5	76.8	66.7	72.3	69.8	70.3
not appropriate (%)	14.9	8.4	11.8	15.4	15.4	20.5	23.2	33.3	27.7	30.2	29.7

**The curfew, i.e. the ban to leave one's own home without a valid reason**											

*n*	*2,988*	*506*	*492*	*502*	*495*	*495*	*498*	–	–	–	–
appropriate (%)	58.5	73.8	66.7	59.1	54.5	49.9	46.6	–	–	–	–
not appropriate (%)	41.5	26.2	33.3	40.9	45.5	50.1	53.4	–	–	–	–

**The mandatory use of masks, i.e. the obligation to wear protective masks in certain situations**											

*n*	*3,021*	–	–	–	–	*501*	*500*	*501*	*510*	*508*	*501*
appropriate (%)	80.4	–	–	–	–	86.4	83.4	81.6	73.1	78.4	79.4
not appropriate (%)	19.6	–	–	–	–	13.6	16.6	18.4	26.9	21.6	20.6
**The distance regulation, i.e. the requirement to maintain a minimum distance of 1.5 metres to other people**											

*n*	*2,025*	–	–	–	–	–	–	*504*	*510*	*504*	*507*
appropriate (%)	89.8	–	–	–	–	–	–	89.7	87.7	91.8	90.1
not appropriate (%)	10.2	–	–	–	–	–	–	10.3	12.3	8.2	9.9

**The limitation of the maximum number of customers in shops**											

*n*	*2,011*	–	–	–	–	–	–	*500*	*508*	*501*	*502*
appropriate (%)	82.6	–	–	–	–	–	–	83.7	80.3	86.2	80.0
not appropriate (%)	17.4	–	–	–	–	–	–	16.3	19.7	13.8	20.0

**The restrictions in day-care centres and schools**											

*n*	*1,458*	–	–	–	–	–	–	–	*504*	*479*	*474*
appropriate (%)	64.3	–	–	–	–	–	–	–	69.0	66.5	56.9
not appropriate (%)	35.7	–	–	–	–	–	–	–	31.0	33.5	43.1

W = wave; only valid responses were included in the analyses.

Table 6 contains information on the respondents’ information behaviour. Respondents were asked how well informed they feel about the situation regarding the coronavirus. Starting in wave 4, this question was asked every other wave to allow the introduction of a new question regarding the evaluation of the media coverage of the coronavirus pandemic. In an open-ended question, respondents were also asked to list the sources they use to inform themselves about what is happening regarding the coronavirus.

**Table 6:** Data on information behaviour.

	Total	W1	W2	W3	W4	W5	W6	W7	W8	W9	W10
**How well or badly do you feel informed about what is happening with the novel coronavirus?**Rating question using a response scale of 1 ‘very bad’ – 5 ‘very good’											

*n*	*3,017*	*510*	*500*	*499*	–	*500*	–	*502*	–	*507*	–
*M*	3.94	4.06	3.96	3.96	–	3.98	–	3.90	–	3.76	–
*SD*	1.17	1.14	1.15	1.26	–	1.10	–	1.13	–	1.19	–

**How do you evaluate the overall media coverage of the novel coronavirus?**Single selection question											

*n*	*1,965*	–	–	–	*491*	–	*480*	–	*492*	–	*502*
downplaying (%)	3.1	–	–	–	2.9	–	2.1	–	4.4	–	3.0
appropriate (%)	59.5	–	–	–	62.5	–	62.5	–	54.3	–	58.8
exaggerated (%)	37.4	–	–	–	34.6	–	35.4	–	41.3	–	38.2

**What sources do you use to inform yourself about what is happening with the novel coronavirus?**Open-ended question											

*n*	*5,003*	*507*	*498*	*502*	*511*	*505*	*491*	*493*	*501*	*493*	*502*
public institutions (%)	6.3	8.6	6.3	6.8	6.8	8.1	5.7	4.2	5.7	7.0	3.7
social environment (%)	8.1	13.1	7.0	8.2	2.9	11.7	4.6	9.3	8.9	8.4	6.6
television (%)	75.0	73.5	79.4	77.6	82.6	74.7	74.2	71.2	73.0	65.1	78.2
radio/podcasts (%)	30.6	29.5	36.4	34.1	29.5	33.4	31.4	25.8	32.4	22.8	30.8
print media (%)	36.6	31.8	34.2	34.0	35.6	33.4	38.4	37.4	43.5	37.0	40.7
internet (%)	61.9	56.9	66.2	61.6	63.0	59.9	61.1	68.4	60.8	65.7	55.8
media in general (%)	7.2	9.5	8.4	9.6	4.2	4.7	4.0	9.2	10.0	8.6	3.8
other (%)	4.8	4.2	3.8	7.4	3.9	6.0	4.0	4.5	4.0	6.5	3.6

W = wave; only valid responses were included in the analyses.

## 2 Experimental Design, Materials and Methods

Data were collected via ten weekly telephone surveys, conducted each Tuesday between 24 March and 26 May 2020 in the Federal Republic of Germany (see Table 1 for an overview). The surveys were conducted by the market research institute Kantar as part of their daily omnibus telephone interviews (computer assisted telephone interviewing, CATI [1]). In an omnibus survey, the market research institute combines several short questionnaires by different clients into one larger survey. The socio-demographic variables are collected only once using a standardized questionnaire by the market research institute, and the data are then made available to each client within their respective data set. A limitation of omnibus surveys is that several questionnaires are combined, and therefore the completion of one questionnaire can bias the responses to the following questionnaires. To still ensure comparability across all waves, our questions were always asked at the same point in the omnibus survey, following a short political questionnaire including the so-called “Sonntagsfrage” (“Sunday question”, a regular question in population surveys regarding the respondent's voting intention).

The statistical population consisted of all German-speaking people aged 14 and over, who could be reached via telephone. The samples were drawn using a random digit dialing procedure that guarantees inclusion of mobile and landline telephone numbers not listed in phonebooks or directories. Over the course of the ten waves, four respondents were excluded since they spontanously claimed during the interview that they had never heard of the coronavirus. Each week, a new, independent sample was drawn. The sample sizes were very similar throughout the waves, ranging from *n* = 500 to *n* = 515 respondents (*N* = 5,076 in total across all waves). If a mobile phone number was dialed, the person who answered the phone was directly selected for the interview. However, a two-stage selection procedure was used for landline telephone numbers. If more than one person aged 14 years or over lived in the respective household, the Kish selection method [2] was utilized to randomly select the respondent. Additionally, to ensure the comparability and representativeness of the collected data, data were statisticially weighted [3]. In a first step, data were weighted regarding the number of mobile phones and landline numbers a person could be reached by to ensure that each person had the same chance to be selected for an interview. In a second step, data were weighted according to sociodemographic variables, including gender, education, age, employment, size of city and German federal state. To guarantee comparability, this weighting procedure was carried out in the exact same way for each individual wave.

An overview of the questionnaire used in the surveys can be found in Appendix A. For closed questions, we used 5-point Likert scales, where appropriate, to increase the variance in our survey results. However, for the question concerning acceptance of governmental measures (see Table 5), we decided to use a binary response format (appropriate vs. not appropriate) as the number of items within this question was quite high and the length of questionaires in the omnibus survey was strictly limited. In addition to closed questions, the questionnaire also contained two open-ended questions: one on the protection measures utilized by the respondents (see Table 3) and one on their sources of information (see Table 4). For both questions, the respondents’ answers were paraphrased and coded. The utilized code frames (Appendix B) were developed based on the paraphrased answers within an inductive process.

Because of the rapidly changing situation with the coronavirus in Germany, the questionnaire underwent several adjustments over the course of the data collection period: Questions or items were replaced or new items were added. As an example, the question about the preferred use of soap or disinfectant was omitted in W4 after showing very similar results in the first three weeks of the survey. This allowed for the inclusion of a new question regarding the perceived impact of the novel coronavirus on one's health versus on one's economic situation – an aspect that gained public interest at that time. Due to rapidly changing regulations concerning the containment of the novel coronavirus in Germany, we also had to continuously update the item list regarding the acceptance of these containment measures. New items were added to incorporoate new regulations (i. e. the mask mandate in W5), and some items were dropped once the regulation was no longer in effect (i. e. the curfew in W7). If regulations were modified, we adjusted the item texts to reflect these changes accordingly. This allowed for a continuous tracking of the public opinion and risk perception regarding the coronavirus pandemic in Germany. All adaptations in the questionnaire are listed in Appendix A.

## Ethics Statement

Ethical approval was not required for this study based on the following considerations: The study did not include medical aspects, person-identifiable data or sensitive or confidential data. No experimental manipulation or psychological tetsts were used. It was always possible for respondents to drop out of the survey before completion or to not answer one or more questions in the survey. In addition, data collection was carried out in line with the standards established by the Association of German Market Research Institutes (ADM; see https://www.adm-ev.de/en/standards-guidelines/). Respondents expressed their consent to participate in the surveys. All data were recorded and processed anonymously.

## CRediT Author Statement

**Fabian Kirsch:** Conceptualization, Methodology, Formal Analysis, Data Management, Writing – Original Draft, Writing – Review & Editing; **Ann-Kathrin Lindemann:** Conceptualization, Methodology, Formal Analysis, Data Management, Writing – Original Draft, Writing – Review & Editing; **Mark Lohmann:** Conceptualization, Supervision; **Gaby-Fleur Böl:** Conceptualization, Supervision.

## Declaration of Competing Interest

The authors declare that they have no known competing financial interests or personal relationships which have or could be perceived to have influenced the work reported in this article.

## Appendix Supplementary materials

Appendix A: Questionnaire Image, application 1

Appendix B: Code frames Image, application 2

Raw data (SPSS) Image, application 3

Raw data (CSV) Image, application 4

Coded data (SPSS) Image, application 5

Coded data (CSV) Image, application 6
