# Supplementary material for: Serial cross-sectional data on the public's perception on the coronavirus during the first months of the pandemic in Germany
Source: Data Brief. 2021 Sep 25;38:107430. doi: 10.1016/j.dib.2021.107430 (PMC8463299; doi:10.1016/j.dib.2021.107430)
Supplement: Appendix B: Code frames [file mmc2.docx]

**Appendix B: Codes frames**

**Question V3**

What sources do you use to inform yourself about what is happening with the novel coronavirus? You can name up to 3 sources.

| **Variable** | **Label** | **Description** | **Exemplary information sources** |
| --- | --- | --- | --- |
| V3_c1 | Public institutions | The source is a public institution. This includes universities, public authorities, research institutions, ministries, government offices as well as more general mentions of the state, federal states, cities or municipalities. | - Robert Koch-Institute (RKI) - Johns-Hopkins-University - health offices - website of the World Health Organisation (WHO) - apps from public institutions |
| V3_c2 | Social environment | The source is a direct, personal contact or interaction with other people or institutions from one’s social environment. This includes personal and professional contacts. | - friends and acquaintances - family - colleagues - conversations - employer - teachers - general practitioner |
| V3_c3 | Television | The source is content produced for television. This includes specific television formats, programmes and channels, even if they are not directly consumed via a television set. | - television/TV - specific television channels - TV news programmes - teletext - broadcast (only in combination with “radio”) |
| V3_c4 | Radio/Podcast | The source is a purely auditory medium. This includes radio and podcasts. | - radio - specific radio stations - specific podcasts - broadcast (only in combination with “television”) |
| V3_c5 | Print media | The source is a printed product. This includes newspapers, journals, brochures, books and magazines. | - press - daily or weekly newspaper - specific newspapers - non-fiction books |
| V3_c6 | Internet | The source is the internet. This also includes specific websites, social media and apps. | - internet - Google - apps - specific news websites - websites and apps of public authorities - Instagram |
| V3_c7 | Media in general | The source is a not more closely defined medium. This also includes mentions that based on their context cannot be clearly assigned to another category. | - media - mass media - news - Broadcasters that broadcast both television and radio programmes - broadcast (without additionally mentioning „television“ or „radio“) |
| V3_c97 | Other | The source cannot be assigned to any of the other categories. This also includes the non-specific naming of devices. | - pharmacy/doctor (when not clear if this was a personal contact) - scientific publications - stores - smartphone - tablet |

The respondents’ answers were paraphrased and coded. The utilized code frame was developed based on the paraphrased answers (inductive process).

All variables are coded as follows:

0 – not mentioned

1 – mentioned

9 – don't know / no answer

**Question V5**

Have or had you taken measures to protect yourself or your family from the novel coronavirus?

| **Variable** | **Label** | **Description** | **Exemplary protection measures** |
| --- | --- | --- | --- |
| V5_c1 | Hygienic measures | The measure describes a hygienic behaviour. This includes the use of disinfectants, washing hands and following hygiene rules in general. | - washing hands - disinfectant - sneezing into one’s elbow - increased hygiene - hygiene recommendations |
| V5_c2 | Protective clothing | The measure refers to the wearing and organising of protective clothing. This particularly includes masks and gloves. | - sewn a mask - mandatory wearing of masks - face mask - gloves |
| V5_c3 | Reduction of contacts | The measure aims at reducing interpersonal contacts. This includes cancelling appointments, staying at home, working from home and avoiding public transport. | - no / limited contacts - quarantine - avoiding the public - staying at home - working from home - avoiding events - cancelling visits - cancelling holiday trips - avoid using public transport - contact only via telephone |
| V5_c4 | Keeping physical distance | The measure aims to maintain physical distance when meeting another person. This also includes avoiding physical contact. | - keeping physical distance - avoid hugging - distance regulation |
| V5_c5 | Adjusted consumer behaviour | The measure describes an adjustment in consumer behaviour. This includes stockpiling, food deliveries and limiting the number of people going shopping. | - stockpiling - grocery delivery - go shopping less frequently - go shopping alone - shop more consciously - go shopping at other times |
| V5_c6 | Compliance with orders and recommendations in general | The measure describes the general compliance with orders and recommendations. This only includes orders and recommendations that are not further specified and cannot be assigned to any other category. | - adhere to guidelines - follow rules - recommended measures - recommendations of the RKI |
| V5_c97 | Other | The measure cannot be assigned to any of the other categories. | - praying - advise/inform others - inform oneself - walks - being mindful of one’s diet - strengthening the immune system - contacts (non-specific) |

The respondents’ answers were paraphrased and coded. The utilized code frame was developed based on the paraphrased answers (inductive process).

All variables are coded as follows:

0 – not mentioned

1 – mentioned

9 – don't know / no answer
